# Supplementary material for: Resolving the full spectrum of human genome variation using Linked-Reads
Source: Genome Res. 2019 Apr;29(4):635–45. doi: 10.1101/gr.234443.118 (PMC6442396; doi:10.1101/gr.234443.118)
Supplement: Supplemental Material [file supp_gr.234443.118_Supplemental_longranger-2.2.zip › longranger-2.2.tar.bz2!/lr2.2-public/lib/stan/cmdstan/stan/doc/api/html/agrad_2fwd_2matrix_2trace__gen__quad__form_8hpp_source.html]

Stan: src/stan/agrad/fwd/matrix/trace\_gen\_quad\_form.hpp Source File


|  |  |
| --- | --- |
|  | Stan  2.5.0  probability, sampling & optimization |


- Main Page
- Related Pages
- Namespaces
- Classes
- Files

- File List
- File Members

All Classes Namespaces Files Functions Variables Typedefs Enumerations Enumerator Friends Macros Pages

- src
- stan
- agrad
- fwd
- matrix

trace\_gen\_quad\_form.hpp

Go to the documentation of this file.

1 #ifndef STAN\_\_AGRAD\_\_FWD\_\_MATRIX\_\_TRACE\_GEN\_QUAD\_FORM\_HPP

2 #define STAN\_\_AGRAD\_\_FWD\_\_MATRIX\_\_TRACE\_GEN\_QUAD\_FORM\_HPP

3

4 #include <stan/math/error\_handling/matrix/check\_multiplicable.hpp>

5 #include <stan/math/error\_handling/matrix/check\_square.hpp>

6 #include <stan/agrad/fwd/matrix/multiply.hpp>

7 #include <stan/math/matrix/trace.hpp>

8 #include <stan/math/matrix/multiply.hpp>

9 #include <stan/math/matrix/transpose.hpp>

10

11 namespace stan {

12  namespace agrad {

13  template<int RD,int CD,int RA,int CA,int RB,int CB,typename T>

14  inline fvar<T>

15  trace\_gen\_quad\_form(const Eigen::Matrix<fvar<T>,RD,CD> &D,

16  const Eigen::Matrix<fvar<T>,RA,CA> &A,

17  const Eigen::Matrix<fvar<T>,RB,CB> &B)

18  {

19  using stan::agrad::multiply;

20  using stan::math::multiply;

21

22  stan::math::check\_square("trace\_gen\_quad\_form(%1%)",A,"A",(double\*)0);

23  stan::math::check\_square("trace\_gen\_quad\_form(%1%)",D,"D",(double\*)0);

24  stan::math::check\_multiplicable("trace\_gen\_quad\_form(%1%)",A,"A",

25  B,"B",(double\*)0);

26  stan::math::check\_multiplicable("trace\_gen\_quad\_form(%1%)",B,"B",

27  D,"D",(double\*)0);

28  return stan::math::trace(multiply(multiply(D,stan::math::transpose(B)),

29  multiply(A,B)));

30  }

31  }

32 }

33

34 #endif

35

check\_multiplicable.hpp

stan::agrad::fvar

**Definition:** fvar.hpp:13

stan::agrad::trace\_gen\_quad\_form

fvar< T > trace\_gen\_quad\_form(const Eigen::Matrix< fvar< T >, RD, CD > &D, const Eigen::Matrix< fvar< T >, RA, CA > &A, const Eigen::Matrix< fvar< T >, RB, CB > &B)

**Definition:** trace\_gen\_quad\_form.hpp:15

stan::agrad::multiply

Eigen::Matrix< fvar< T >, R1, C1 > multiply(const Eigen::Matrix< fvar< T >, R1, C1 > &m, const fvar< T > &c)

**Definition:** multiply.hpp:21

transpose.hpp

trace.hpp

stan::math::check\_multiplicable

bool check\_multiplicable(const char \*function, const T1 &y1, const char \*name1, const T2 &y2, const char \*name2, T\_result \*result)

**Definition:** check\_multiplicable.hpp:15

stan::math::transpose

Eigen::Matrix< T, C, R > transpose(const Eigen::Matrix< T, R, C > &m)

**Definition:** transpose.hpp:12

multiply.hpp

stan::math::trace

T trace(const Eigen::Matrix< T, Eigen::Dynamic, Eigen::Dynamic > &m)

Returns the trace of the specified matrix.

**Definition:** trace.hpp:20

stan::math::multiply

boost::enable\_if\_c< boost::is\_arithmetic< T >::value, Eigen::Matrix< double, R, C > >::type multiply(const Eigen::Matrix< double, R, C > &m, T c)

Return specified matrix multiplied by specified scalar.

**Definition:** multiply.hpp:25

multiply.hpp

stan::math::check\_square

bool check\_square(const char \*function, const Eigen::Matrix< T\_y, Eigen::Dynamic, Eigen::Dynamic > &y, const char \*name, T\_result \*result)

Return true if the specified matrix is square.

**Definition:** check\_square.hpp:24

check\_square.hpp


---

[ Stan Home Page ]

*© 2011–2014,
Stan Development Team.*
